# Supplementary material for: Mec-Positive Staphylococcus Healthcare-Associated Infections Presenting High Transmission Risks for Antimicrobial-Resistant Strains in an Equine Hospital
Source: Antibiotics (Basel). 2022 May 4;11(5):621. doi: 10.3390/antibiotics11050621 (PMC9137905; doi:10.3390/antibiotics11050621)
Supplement: Supplementary file 1 [file antibiotics-11-00621-s001.zip › antibiotics-1687384-SM.pdf]

Supplementary Material A. Results detail

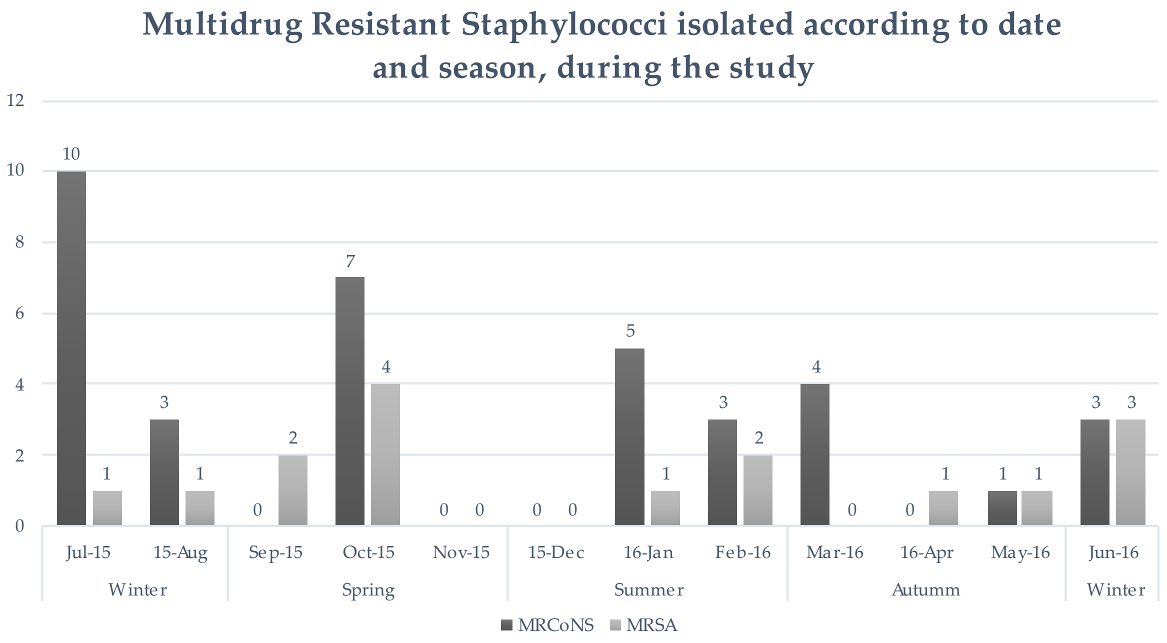

Figure S1. Number of isolates of MRSA obtained monthly during this study. The "x" axis represents each month during the year period, and the season of the year. The "y" axis represents the number of isolates obtained. The months where no isolates are specified, is because no suggestive isolate was obtained in the microbiological procedure, or because no *mec*A nor *mec*C gene could be amplified (Nov-15; one suggestive isolate; Dec-15, no suggestive isolate), or because the isolates obtained could not be isolated from other contaminant bacteria, like *Pseudomonas* spp. (Mar-16; one isolate), therefore they were discarded from the study.

Table S1. Percent Nucleotide Identity Matrix was obtained using Clustal Omega 2.1.

	MRSA-PS-02	*MRSA*-PS-08	*MRSA*-PS-12
MRSA-PS-02	100%	97.8%	100%
MRSA-PS-08	97.8%	100%	97.8%
MRSA-PS-12	100%	97.8%	100%

	S. aureus	CoNS	S. aureus or CoNS	S. aureus AND CoNS	Ambiental	patient	Human	Animal	Comun	Autumn	Spring	Winter	Summer	Equipment	Outside	Proceedings	Surgery	Hospitalization
**S. aureus**	1.00	0.31	0.58	0.70	-0.05	0.05	0.00	0.05	-0.04	-0.03	0.01	0.03	-0.01	-0.02	-0.02	0.04	0.04	-0.05
**CoNS**	0.31	1.00	0.90	0.45	-0.07	0.07	-0.01	0.06	-0.04	-0.04	-0.03	0.09	-0.02	-0.07	-0.02	0.02	-0.01	-0.07
**S. aureus or CoNS**	0.58	0.90	1.00	0.41	-0.08	0.08	-0.01	0.08	-0.06	-0.04	-0.01	0.09	-0.03	-0.06	-0.04	0.01	0.01	-0.06
**S. aureus AND CoNS**	0.70	0.45	0.41	1.00	-0.03	0.03	0.00	0.01	-0.01	-0.02	-0.02	0.04	0.00	-0.03	0.00	0.09	0.01	-0.07
Ambiental	-0.05	-0.07	-0.08	-0.03	1.00	-1.00	0.24	-0.99	0.70	0.07	-0.03	0.04	-0.07	0.21	0.21	0.20	0.28	-0.64
patient	0.05	0.07	0.08	0.03	-1.00	1.00	-0.24	0.99	-0.70	-0.07	0.03	-0.04	0.07	-0.21	-0.21	-0.20	-0.28	0.64
Human	0.00	-0.01	-0.01	0.00	0.24	-0.24	1.00	-0.24	-0.51	0.05	-0.01	-0.01	-0.02	-0.15	-0.15	0.39	0.13	-0.14
Animal	0.05	0.06	0.08	0.01	-0.99	0.99	-0.24	1.00	-0.71	-0.06	0.03	-0.04	0.07	-0.21	-0.21	-0.20	-0.29	0.64
Comun	-0.04	-0.04	-0.06	-0.01	0.70	-0.70	-0.51	-0.71	1.00	0.01	-0.01	0.05	-0.04	0.29	0.30	-0.11	0.17	-0.47
Autumn	-0.03	-0.04	-0.04	-0.02	0.07	-0.07	0.05	-0.06	0.01	1.00	-0.33	-0.31	-0.34	-0.02	0.08	-0.03	0.04	-0.06
Spring	0.01	-0.03	-0.01	-0.02	-0.03	0.03	-0.01	0.03	-0.01	-0.33	1.00	-0.33	-0.35	0.00	-0.07	0.01	0.04	0.03
Winter	0.03	0.09	0.09	0.04	0.04	-0.04	-0.01	-0.04	0.05	-0.31	-0.33	1.00	-0.34	0.03	0.05	0.02	-0.06	-0.02
Summer	-0.01	-0.02	-0.03	0.00	-0.07	0.07	-0.02	0.07	-0.04	-0.34	-0.35	-0.34	1.00	-0.01	-0.05	0.00	-0.01	0.05
Equipment	-0.02	-0.07	-0.06	-0.03	0.21	-0.21	-0.15	-0.21	0.29	-0.02	0.00	0.03	-0.01	1.00	-0.14	-0.13	-0.20	-0.30
Outside	-0.02	-0.02	-0.04	0.00	0.21	-0.21	-0.15	-0.21	0.30	0.08	-0.07	0.05	-0.05	-0.14	1.00	-0.14	-0.21	-0.32
Proceedings	0.04	0.02	0.01	0.09	0.20	-0.20	0.39	-0.20	-0.11	-0.03	0.01	0.02	0.00	-0.13	-0.14	1.00	-0.19	-0.29
Surgery	0.04	-0.01	0.01	0.01	0.28	-0.28	0.13	-0.29	0.17	0.04	0.04	-0.06	-0.01	-0.20	-0.21	-0.19	1.00	-0.44
Hospitalization	-0.05	-0.07	-0.06	-0.07	-0.64	0.64	-0.14	0.64	-0.47	-0.06	0.03	-0.02	0.05	-0.30	-0.32	-0.29	-0.44	1.00

Figure S2. Bivariate correlation table using Pearson's coefficients (N=979 observations). Darker colors indicate negative correlation whereas lighter positive correlation.

Table S2. Univariate logistic analysis (N=979 observations)

Variables/Models	I.			II.			III.			IV.		
	MRSA			MRCoNS			MRSA or MRCoNS			MRSA and MRCoNS		
	OR	95% CI	P-value	OR	95% CI	P-value	OR	95% CI	P-value	OR	95% CI	P-value
Origen												
**Environmental**	0.44	0.15,1.28	0.13	0.44**	0.22,0.89	0.02	0.44**	0.23,0.83	0.01	0.44	0.10,2.00	0.29
Patient	2.27	0.78,6.60	0.13	2.27**	1.12,4.59	0.02	2.29**	1.20,4.36	0.01	2.25	0.50,10.12	0.29
Use type												
Human	0.96	0.21,4.33	0.96	0.79	0.27,2.27	0.66	0.82	0.31,2.12	0.67	0.96	0.11,8.02	0.97
**Animal**	2.27	0.78,6.60	0.13	1.99*	0.97,4.06	0.06	2.29**	1.20,4.36	0.01	1.19	0.23,6.19	0.83
Common	0.49	0.17,1.43	0.19	0.62	0.31,1.23	0.17	0.53*	0.28,1.00	0.05	0.89	0.20,3.98	0.87
Season												
Autumn	0.54	0.12,2.42	0.42	0.57	0.22,1.50	0.26	0.56	0.23,1.36	0.20	0.54	0.06,4.51	0.57
Spring	1.16	0.36,3.74	0.80	0.64	0.26,1.56	0.32	0.84	0.39,1.78	0.64	0.48	0.06,4.02	0.50
**Winter**	1.77	0.59,5.32	0.31	2.73***	1.36,5.51	0.00	2.43***	1.28,4.63	0.01	2.38	0.53,10.71	0.26
Summer	0.74	0.21,2.69	0.65	0.73	0.31,1.70	0.47	0.67	0.31,1.48	0.33	1.10	0.21,5.68	0.91
Area of the hospital												
Equipment	0.57	0.07,4.39	0.59	1.00	1.00,1.00	1.00	0.18*	0.03,1.35	0.10	1.00	1.00,1.00	1.00
Outside	0.5	0.06,3.84	0.50	0.64	0.19,2.14	0.47	0.52	0.16,1.71	0.28	1.09	0.13,9.12	0.94
Proceedings	2.23	0.61,8.14	0.22	1.46	0.55,3.87	0.44	1.16	0.44,3.03	0.76	6.19 **	1.37,28.05	0.02
Surgery	1.93	0.64,5.81	0.24	0.92	0.39,2.15	0.85	1.15	0.55,2.39	0.71	1.38	0.27,7.14	0.70
**Hospitalization**	0.4	0.11,1.45	0.16	0.39**	0.17,0.91	0.03	0.48*	0.23,1.00	0.05	1.00	1.00,1.00	1.00

Notes: * p<0.1, ** p<0.05, *** p<0.01. Robust standard errors were used.
Wald and Likelihood ratio tests were employed, and non-statistically significant values were found for other than the independent variables selected from the univariate logistic regression model at the p-value=0.1 level'

Table S3. Multivariate logistic analysis (N=979 observations) $OR_{ModelIII}$

Variables/Models	I. Drug-resistant *S. aureus*			II. Drug-resistant *CoNS*			III. Drug resistant *S. aureus* OR Drug-resistant *CoNS*			IV. Drug-resistant *S. aureus* AND Drug-resistant *CoNS*		
	OR	95% CI	P-value	OR	95% CI	P-value	OR	95% CI	P-value	OR	95% CI	P-value
Origen (Ref.: Patient)												
Environmental	0.21	0.00,18e+6	0.87	0.02***	0.00,0.31	<0.001	0.17	0.00,141.54	0.61	0.00***	0.00,0.10	<0.001
Use type (Ref.: Human and common)												
Animal	4.8	0.00,4.30e+8	0.87	0.69	0.03,14.36	0.81	5.85	0.01,4838.93	0.61	0.13	0.00,3.47	0.22
Season (Autumn, Spring or Summer)												
Winter	1.72	0.53,5.54	0.37	3.05***	1.50,6.21	<0.001	2.62***	1.38,4.98	<0.001	2.84	0.54,15.06	0.22
Area of the hospital (Ref.: Equipment, Outside, Surgery)												
Hospitalization	0.04*	0.00,0.55	0.02	0.03***	0.00,0.25	<0.001	0.03***	0.00,0.31	<0.001	1.00	1.00,1.00	1.00
Constant	0.06	0.00,51e+5	0.76	1.06	0.08,14.65	0.96	0.17	0.00,141.42	0.61	1.00	0.06,15.95	1.00
AIC		139.58			251.29			296.5			67.78	
Model significance (p-value)		0.02			<0.001			<0.001			<0.001	

Notes: * p<0.05, ** p<0.01, *** p<0.001. Robust standard errors were used.

Supplementary Material B. Methodology detail

Sample procedure

We obtained, at least, four samples per patient. These samples included nostrils (both), armpits (both), and wounds (including surgical incisions), in case there was any [35]. All the samples were maintained at 4°C during sampling and immediately transferred to the laboratory at Universidad Andres Bello (Santiago, Chile) for further analysis.

Staphylococcus spp. isolation and identification

MALDI TOF analysis, one colony of each sample was applied to the metal plate and mixed with HCCA Matrix (Bruker, Massachusetts, USA). Then, the plate was put into the MALDI Biotyper (Bruker, Massachusetts, USA). The spectrum generated was instantly matched against the reference library by the MALDI Biotyper CA. System Software (Bruker, Massachusetts, USA).

Each sample was streaked in ORSA and cultured at 37°C overnight. Blue colonies on colorless media were presumptive of oxacillin resistant staphylococci. All suspected colonies were then streaked in Mannitol Salt Agar (MSA) (BD, Franklin Lakes, NJ) and Baird Parker Agar with Rabbit Plasma Fibrinogen (BP+RPF) (Biomérieux, Marcy-l'Étoile, France) [50], cultured at 37°C for 48 hours and checked for mannitol fermentation and coagulase activity, respectively. After that, all suspected colonies were transferred to Tryptic Soy Agar (TSA) (BD, Franklin Lakes, NJ) and cultured at 37°C overnight for further characterization.

Antimicrobial susceptibility.

Between 5-8 colonies were suspended in 5 ml of Müller Hinton broth (BD, Franklin Lakes, NJ) and incubated at 37°C overnight. Cultures were adjusted to $1.5x10^{8}$ CFU/mL using a McFarland 0.5 standard (BioMérieux, France), and streaked on Müller Hinton agar. Experiments were conducted in two independent duplicates. The antimicrobials used were azithromycin (15 ug) (AZM), cefoxitin (30 ug) (FOX), ciprofloxacin (5 ug) (CIP), clindamycin (2 ug) (CLI), chloramphenicol (30 ug) (CHL), gentamicin (10 ug) (GEN), linezolid (30 ug) (LZD), oxacillin (1 ug) (OXA), penicillin (10 units) (PEN), rifampicin (5 ug) (RIF), trimethoprim/sulfamethoxazole (1:19 25 ug) (SXT), tetracycline (30 ug) (TET), OXOID™ (Hampshire, UK) (NARMS panel, FDA 2020) [44, 51]

Detection and confirmation of methicillin-resistance *mecA* and *mecC*.

The amplifications were performed on supernatants from crude DNA extracts according to the methods previously described [4]. The primers and PCR conditions used have also been described and published elsewhere [16, 30, 31] (Table 1). Positive isolates for *mec*A or *mec*C, were immersed in a 30% solution of glycerol (Winker, Santiago, Chile) and Trypticase Soy Broth (TSB) (BD, Franklin Lakes, NJ), and then stored at -80°C.

Confirmation of MRSA with clinical relevance was directed by PCR according to Stegger et al. [27], and amplicon sequencing by Sanger technologies at MACROGEN™ (Korea) considering three isolates MRSA-PS-02, MRSA-PS-08 and MRSA-PS-12. For this were using *mec*A$_{LGA251}$MultiFP and *mec*A$_{LGA251}$RP primers, to three isolates selected randomly (MRSA-PS-02, MRSA-PS-08 and MRSA-

PS-12). This scheme amplified an expected 720 bp amplicon of *mec*C, described for SCC*mec* characterization [31].

Table S4. Primers and PCR conditions used in this study.

Gene	Primer Sequence 5′ – 3′	Amplicon (bp)	Reference
*mec*A[a]	F-TCC AGA TTA CAA CTT CAC CAG G<br>R-CCA CTT CAT ATC TTG TAA CG	162	(Stegger et al., 2011)
*mec*C[b]	F-TCA CCA GGT TCA AC[Y] CAA AA<br>R-CCT GAA TC[W] GCT AAT AAT ATT TC	356	(García-Álvarez et al., 2011)
mecCMulti[c]	F-GAA AAA AAG GCT TAG AAC GCC TC<br>R-CCT GAA TC[W] GCT AAT AAT ATT TC	138	(Stegger et al., 2011)
lukF-pv[a]	F-GCT GGA CAA AAC TTC TTG GAA TAT<br>R-GAT AGG ACA CCA ATA AAT TCT GGA TTG	83	(Stegger et al., 2011)
spa[a]	F-TAA AGA CGA TCC TTC GGT GAG C<br>R-CAG CAG TAG TGC CGT TTG CTT	180 - 600	(Stegger et al., 2011)
nuc[d]	F-GCG ATT GAT GGT GAT ACG GTI<br>R-AGC CAA GCC TTG ACG AAC TAA AGC	267	(Brakstad et al., 1992)

[a] PCR conditions: 94°C x 15 min., 30 cycles (94°C x 30 secs., 59°C x 1 min, and 72°C x 1 min.), 72°C x 10 min.
[b] PCR conditions: 94°C x 5 min; 30 cycles (94°C x 1 min, 55°C x 1 min, and 72°C x 2 min.), 72°C x 5 min.
[c] PCR conditions: 94°C x 15 min., 35 cycles (94°C x 30 secs., 50°C x 1 min, and 72°C x 1 min.), 72°C x 10 min.
[d] PCR conditions: 94°C x 15 min., 37 cycles (94°C x 1 min., 55°C x 30 secs, and 72°C x 1,5 min.), 72°C x 3,5 min.

Table S5. Percent Identity Matrix

	SCoP-02	SCop-08	SCoP-12
SCoP-02	100%	97.8%	100%
SCop-08	97.8%	100%	97.8%
SCoP-12	100%	97.8%	100%

Subtyping by PFGE of strains of *S. aureus*

Strains were grown in Columbia agar plates supplemented with 5% sheep blood and incubated at 37°C for 18-24 h, under aerobic conditions. Selected colonies were suspended in 3 mL of suspension buffer (10 mM Tris; 0.1 mM EDTA; pH 8.0), and the optical density was adjusted to 2-2.5 at 660 nm using an EPOCH spectrophotometer (BioTek®, USA). Subsequently, 300 µL of suspension was transferred to an Eppendorf tube containing 5 µL of lysostaphin [1 mg/mL] (Sigma-Aldrich, Saint Louis, MO, USA) and incubated at 37°C for 10 min. Then, 300 µL of 1% SeaKem Gold agarose (Lonza, Basel, Switzerland) in buffer TE (10 mM Tris EDTA, pH 8.0) was added, and the suspensions were gently mixed. From each mixture, 300 µL were pipetted into plug molds. Agarose plugs were allowed to solidify at room temperature for 5 min. Bacterial lysis was carried out transferring the plugs to centrifuge tubes of 50 mL containing 2 mL of ST lysis buffer (6 mM Tris HCl, 1 M NaCl, 100 mM EDTA, 0.5% Brij-58, 0.2% sodium deoxycholate, 0.5% sodium lauroyl-sarcosine) added with 15 µL of lysozyme [10 mg/mL] (Sigma-Aldrich, Saint Louis, MO, United States), and incubated for 2 h at 37°C. Then it was washed with sterile distilled water preheated to 54°C and 2.5 mL of lysis buffer and 24 µL of proteinase K [100 µg/mL], were added and incubated at 54°C for 2 h under constant agitation

at 150-175 rpm. Afterward, plugs were washed 3 times with 5 mL of MilliQ water at 54°C for 10-15 min with agitation at 150-175 rpm. Three additional washes with 5 mL of TE buffer were performed, and the plugs were stored in 8 mL of TE buffer at 4°C. A plug slice was cut (1/3 of size) and equilibrated in 100 μL of 1:10 restriction buffer for 15 min at 25°C. Then, the buffer was removed and 100 μL of the buffer with *Sma*I enzyme (Promega, Madison, WI, United States) was added, and incubated for 4 h at 25°C. The digestion mix was then removed and 100 μL of TE buffer was added, and it was stored at 4°C. A SeaKem Gold 1% (wt/vol) agarose gel was prepared in 0.5× TBE buffer with 3 μL de GelRed® (Biotium, USA) and the PFGE was performed under the following conditions: 14°C, 6 V/cm, 19 h run-time, 5.3 to 34.9 s switch times. Gel was visualized using a UV transilluminator (UVItec system, Cambridge, UK).
Gel was photographed and digitized with the UVIDOC HD5 Gel Documentation System (Uvitec, Cambridge) and saved as a TIFF file and analyzed with BioNumerics® 6.6 software (AppliedMaths, Inc., Austin, TX, USA). The PFGE pattern homology dendrogram was built using the Dice coefficient. Band position tolerance and optimization were set at 1% and 0.5%, respectively. A similarity coefficient of 80% was selected to define a group as closely related [52].

Statistical analyses

For multivariate models, we included source of the samples (environmental or patient), type of use (common or human contact surface), season (e.g., winter), and area of the hospital where samples were collected (e.g., hospitalization, surgery, etc.) as independent variables after testing them separately in the univariate models (p-value<0.1). All statistical analyses were conducted using Stata 15 software, MP version 2017, StataCorp LLC., College Station, TX, USA.
